# Interobserver variation in cervical cancer tumor delineation for image‐based radiotherapy planning among and within different specialties

**DOI:** 10.1120/jacmp.v6i4.2117

**Published:** 2005-11-22

**Authors:** Dee H. Wu, Nina A. Mayr, Yasemin Karatas, Rifat Karatas, Mustafa Adli, Susan M. Edwards, James D. Wolff, Allen Movahed, Joseph F. Montebello, William T.C. Yuh

**Affiliations:** ^1^ Radiological Sciences University of Oklahoma Oklahoma City Oklahoma; ^2^ Radiation Oncology The Ohio State University Columbus Ohio U.S.A.; ^3^ Diagnostic Radiology The Ohio State University Columbus Ohio U.S.A.

**Keywords:** tumor delineation, magnetic resonance imaging, cervical cancer, radiation therapy

## Abstract

Radiation therapy for cervical cancer involves a team of specialists, including diagnostic radiologists (DRs), radiation oncologists (ROs), and medical physicists (MPs), to optimize imaging‐based radiation therapy planning. The purpose of the study was to investigate the interobserver variations in tumor delineation on MR images of cervical cancer within the same and among different specialties. Twenty MRI cervical cancer studies were independently reviewed by two DRs, two ROs, and two MPs. For every study, each specialist contoured the tumor regions of interest (ROIs) on T2‐weighted Turbo Spin Echo sagittal images on all slices containing tumor, and the total tumor volume was computed for statistical analysis. Analysis of variance (ANOVA) was used to compare the differences in tumor volume delineation among the observers. A graph of all tumor‐delineated volumes was generated, and differences between the maximum and minimum volumes over all the readers for each patient dataset were computed. Challenges during the evaluation process for tumor delineation were recorded for each specialist. Interobserver variations of delineated tumor volumes were significant (p<0.01) among all observers based on a repeated measures ANOVA, which produced an F(5,95)=3.55. The median difference between the maximum delineated volume and minimum delineated volume was 33.5 cm^3^ (which can be approximated by a sphere of 4.0 cm diameter) across all 20 patients. Challenges noted for tumor delineation included the following: (1) partial voluming by parametrial fat at the periphery of the uterus; (2) extension of the tumor into parametrial space; (3) similar signal intensity of structures proximal to the tumor such as ovaries, muscles, bladder wall, bowel loops, and pubic symphysis; (4) postradiation changes such as heterogeneity and necrosis; (5) susceptibility artifacts from bowels and vaginal tampons; (6) presence of other pathologies such as atypical myoma; (7) factors that affect pelvic anatomy, including the degree of bladder distension, bowel interposition, uterine malposition, retroversion, and descensus. Our limited study indicates significant interobserver variation in tumor delineation. Despite rapid progress in technology, which has improved the resolution and precision of image acquisition and the delivery of radiotherapy to the millimeter level, such “human” variations (at the centimeter level) may overshadow the gain from technical advancement and impact treatment planning. Strategies of standardization and training in tumor delineation need to be developed.

PACS number(s):

## I. INTRODUCTION

Tumor delineation is an essential step in the process of radiation therapy. At our institution, critical identification of cervical cancer tumor location is discussed among diagnostic radiologists (DRs), radiation oncologists (ROs), and medical physicists (MPs) in an effort to optimize final placement of radiation therapy target volumes. Accuracy in tumor delineation is believed to improve precision in the radiation therapy process, which leads to more effective treatment and reduced complications.^(1)^

Interobserver variation has been previously reported to demonstrate a variation of up to 13% on CT images in cervical cancer brachytherapy applications^(2)^; however, to date there is not a sufficient quantitative record of magnetic resonance (MR) cervical cancer tumor delineation from different medical subspecialty groups and individuals. Tumor delineation has been reported to have an essential role in the evaluation of the success of radiotherapy treatment.^(3)^ In addition, large‐scale MR perfusion imaging‐based predictive assays for radiation therapy effectiveness are critically based on the ability to provide accurate tumor delineation.^(4)^

To analyze tumor delineation differences, we use specialized in‐house software capable of preserving region‐of‐interest (ROI) information in cervical cancer patients at various stages of treatment and by different individuals and between groups of subspecialists. With six different evaluators (two observers from each subspecialty: RO, DR, MP), we have sought to evaluate whether the level of differences between the groups results in significant volumetric differences. Despite continued advancement in imaging and therapy technology on the order of improvement on the millimeter level, we wanted to estimate whether the variation in tumor delineation sizes of individual evaluators from various subspecialties was of the same magnitude or larger than on the order of millimeters. In addition, as a part of the evaluation process, we also noted heuristic reasons for differences and pitfalls in the tumor delineation process. Such information is intended to benefit clinicians and researchers in the cervical cancer tumor delineation process.

## II. MATERIALS AND METHODS

**Figure 1:**
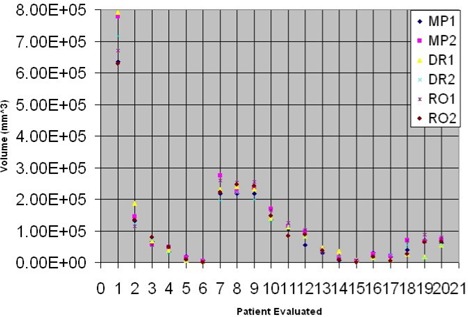
Volumetric differences are illustrated graphically for different delineators across 20 cervical cancer patients.

Twenty MRI cervical cancer studies were independently reviewed by two DRs, two ROs, and two MPs (IRB #09643). For every study, each specialist contoured the tumor ROIs on T2‐weighted Turbo Spin Echo sagittal images on all slices containing tumor. An image viewer using MATLAB (Natick, MA) with compiler toolbox and C/C++ modules was constructed in our lab to permit easy comparison of cervical cancer patient delineation and image review after the analysis and statistical comparison. The tumor was contoured on all acquired slices, and the total tumor volume was computed for statistical analysis. Analysis of variance (ANOVA) was used to compare the differences in tumor volume delineation between the observers. A graph of all tumor‐delineated volumes was generated, and differences between the maximum and minimum volumes over all the readers for each patient dataset were computed (Fig. 1). Challenges during the evaluation process for tumor delineation were recorded for each specialist.

All analysis was performed using SAS release 8.01 (Cary, NC). Statistical analysis was evaluated by the general linear model (GLM). The ANOVA was designed as a repeated measures test across readers; classification by reader and patient was used; and a Tukey honest significant differences (HSD) test was used to classify the readers on a post hoc contrast test in addition to between the three combinations of groups: DR‐MP, DR‐RO, and RO‐MP.

## III. RESULTS

**Figure 2:**
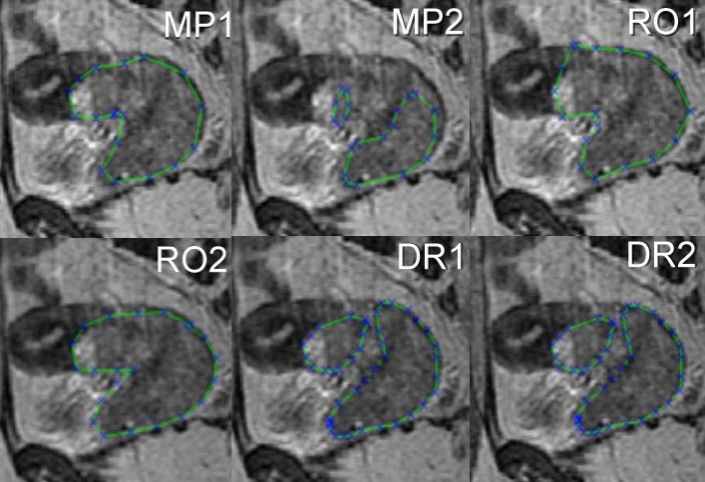
A side‐by‐side comparison of the differences between six different subspecialist delineators is shown on just a single slice from a cervical cancer patient (extracted from up to 40 slices acquired).

Interobserver variations of delineated tumor volumes were significant (p<0.01) among all observers based on a repeated measures ANOVA, which produced an F(5,95)=2.84. The median difference across all individuals between the maximum delineated volume and minimum delineated volume for each patient was 41.4 cm^3^ (equivalent to a sphere of 4.3 cm diameter) across all 20 patients (see Fig. 1). Up to 40 slices for each patient needed to be reviewed by each delineator, making this process very time consuming (see Fig. 2). No differences were found between any of the subspecialist groups (ROs, DRs, and MPs) on the Tukey HSD contrast test. Challenges noted for tumor delineation included the following:

1. partial voluming by parametrial fat at the periphery of the uterus;
2. extension of the tumor into parametrial space;
3. similar signal intensity of structures proximal to the tumor, such as ovaries, muscles, bladder wall, bowel loops, and pubic symphysis;
4. postradiation changes, such as heterogeneity and necrosis;
5. susceptibility artifacts from bowels and vaginal tampons;
6. presence of other pathologies, such as atypical myoma;
7. factors that affect pelvic anatomy, including the degree of bladder distension, bowel interposition, uterine malposition, retroversion, and descensus.

## IV. DISCUSSION AND CONCLUSION

Our limited study indicates significant interobserver variation in tumor delineation. Despite rapid progress in technology, which has improved the resolution and precision of image acquisition and the delivery of radiotherapy to the millimeter level, such “human” variations (at the centimeter level) may overshadow the gain from technical advancement and impact treatment planning. Median differences were on the order of the volume of a 4.3‐cm diameter sphere, much larger than the resolution of the imaging device and the precision of radiation beam placement on a daily basis. No differences were detected between the different subspecialists, suggesting that the differences are based more on individual delineator perception than role in the treatment process.

At our facility, there are only two subspecialists who perform the primary evaluation for cervical MR imaging in each of our DR and RO group. We believe this to be generally true of most U.S.‐based institutions, which are likely to have similar staffing (e.g., there are only two DRs who read all the MR body images at our institution, despite the fact that it covers over 800 beds). To compare between different groups (subspecialists), it would be preferable to have more readers in each subspecialty group. Thus the results presented in this work, which describe between different subspecialist groups, are statistically limited.

It is possible that web‐based software may be created in the future, enabling more participants from different facilities. This would mean an adequate number of personnel per subspecialty could participate. This would improve the statistical validity associated with between‐group comparisons, which is a weakness of this study.

Comfort level with delineation appeared to be dependent on the amount of practice with the particular anatomy because there are many possibilities for pitfalls in the cervix region. The developed software allows the user to save a retrospective review of the tumor volumes for multiple individual delineators. For example, in one case (patient #19), the largest area was $8.8\times 10^{4}\;{\text{cm}}^{3}$. Upon retrospective review, radiation oncologist one (RO1) appears to have included delineated areas that were interpreted as parametrial fat by diagnostic radiologist one (DR1), who excluded these areas and generated a volume nearly six times smaller, at $1.4\times 10^{4}\;{\text{cm}}^{3}$. These results suggest that strategies of standardization and training in tumor delineation should be developed. In addition, when possible, review and discussion of the tumor delineation by all medical subspecialists participating in the patient's treatment process are recommended because of the large level of variation in tumor delineation across individuals.
